# Cancer of the Cervix Uteri

**Published:** 1894-02-24

**Authors:** James Oliver

**Affiliations:** Physician to the Hospital for Women, London


### CANCER OF THE CERVIX UTERI.

By James Oliver, M.D., F.L.S., F.R.S.Edin., Physician to the Hospital for Women, London.

In treating of cancer of the womb it is necessary to divide the cases into two groups, according as the disease originates [in the] cervix or in the body of the organ. Anatomically and physiologically, these two portions—the transmitting and incubating parts—differ from each other, hence it is not astonishing that when attacked by malignant disease they should respectively display, in a more or less marked manner, the tendency to behave as though they were independent organs. If the disease begins in the cervix, it seldom happens that at the time of death the body of the uterus is found to be extensively involved. So, too, when the disease originates in the body of the organ, this portion may become transformed into a malignant mass, without the cervix participating to any marked extent in the change. In a few rare instances, as in Case 2, we may even find these two portions of the same organ occupied by growths of a totally different character.

In the present paper we shall confine our attention to the study of those cases in which the disease originates in the cervix, and shall on a future occasion deal with that group in which it appears primarily in the body of the uterus.

In the majority of the cases of cancer of the cervix, the disease begins apparently in the region of the external os, and especially in its posterior lip, but with greater or less rapidity the epithelial cells infiltrate the whole of this portion of the uterus, and convert it into an irregularly nodulated mass. Sooner or later, in consequence of a direct continuity of tissue, the mucous membrane which enters into the formation of the vaginal roof becomes similarly involved, and, as a rule, it is evident that the disease has progressed more in the direction of the posterior than of the anterior fornix. Very soon ulceration of the surface of the growth takes place, but by degrees this process extends more deeply, and eventually serious hæmorrhage may result from large vessels being thereby opened. Sometimes the morbid cells, showing—at least for a time—but a feeble disposition to penetrate the tissues, proliferate more superficially, and form an excrescence which fills more or less completely the vagina, and resembles a cauliflower growth. In a few rare cases the disease begins as a superficial ulcer, the margins of which are irregular, red, and irritable-looking, but not at all indurated. Eventually, however, and somewhat rapidly, by a process of liquefaction, the base of this ulcer becomes excavated, and the surrounding tissues become indurated.

The importance of the influence of heredity in the causation of cancer is not now so universally allowed as it was, yet it must nevertheless be evident that it is impossible to formulate any conclusion regarding this question solely by the aid of ordinary statistics. The tendency to a disease may be inherited and transmitted, and yet the actual evolution of the disease may take place after one or more generations have experienced immunity. For the evolution of every natural phenomenon certain conditions are necessary, but we are often astounded when we learn how insignificant apparently is the influence which prevents a more or less sudden transformation, or which preserves the potential state.

Cancer of the cervix is an important disease, not only on account of its nature and frequency, but also on account of the insidious manner in which it so often develops. It may develop during pregnancy, as in Case 1, or a woman may become pregnant soon after the disease has attacked the cervix. The discharge from a cancerous growth kills the spermatozoa, so that in many cases impregnation is thus prevented.

The disease originates most commonly between the ages of thirty-five and forty-five. Frequently it is observed after the cessation of the menopause, and in a few rare cases it may occur before the age of thirty, as in Case 2.

Women who have borne children, and those especially who have had large families, are more liable to suffer from this disease than those who never have been pregnant. Women who live in celibacy, however, are not exempt.

*Symptoms.*—The commonest symptom is hæmorrhage, which is usually profuse and more or less constant. Discharge is an invariable association of malignant disease of the cervix. It is often watery, and sooner or later it becomes offensive. Pain is in the majority of cases a late symptom. It is usually worse towards evening, and is often referred to the hips and outside of thighs. Disturbance of the bladder is rarely complained of until the disease is far advanced. Occasionally the bladder or rectum, or both viscera, are eaten into, and urine or fæces, or both, may pass *per vaginam.*

It is impossible to state approximately the probable duration of life in any given case of cancer of the cervix. If the disease is in existence during pregnancy it tends to run a rapid course. Reckoning from the time when definite symptoms of the disease were observed, I have seen cases in which cancer of the cervix proved fatal after having existed for three or four years. It is, of course, impossible to say how long the disease may exist before it produces any symptom.

*Diagnosis.*—In the majority of the cases the disease is well advanced before the patient seeks advice, and the condition is then very evident. The roof of the vagina may be occupied by an irregular and nodulated mass, which may have ulcerated more or less extensively, or the vagina may contain a vegetating mass which is friable and presents the characters of a cauliflower excrescence. Usually a malignant growth bleeds readily when touched. In some cases it may be necessary to call in the aid of the sense of sight, and to obtain a good view of the cervix a large-sized Fergusson's speculum should be employed.

*Treatment.*—If after careful examination it is evident that the disease has not extended to the vaginal roof, then simple or supra-vaginal amputation of the cervix may be recommended. If supra-vaginal amputation is not feasible, then total extirpation of the uterus is unwarrantable. The former is an operation which involves but little risk when compared with the latter, and it is highly probable that the possibility of immunity from, or recurrence of the disease, is the same for both. I have seen cases in which after simple and supra-vaginal amputation there was no recurrence of the disease after a lapse of seven years. If the disease is too for advanced to justify operative interference, then symptoms should be treated. If hæmorrhage is troublesome an injection of perchloride of iron may prove useful. For pain morphia suppositories may be recommended. In some cases the symptoms of cancer of the cervix are mitigated by the internal administration of Chian turpentine, but the drug produces no change in the growth.

Case 1.—Epithelioma of cervix uteri, developing during pregnancy, and proving rapidly fatal:—

Elizabeth M., æt. 34, and married eleven years, has had six children. The last child was born on July 7th, 1892. For two months before this confinement patient had complained of a bloody discharge, which at times became watery. Since the confinement there has been constantly a discharge of blood from the vagina. On September 7th, 1892, when I examined this patient, the vaginal roof was occupied by an irregularly nodulated mass, the centre of which was extensively ulcerated. She died on September 28th, 1892.

In this case hæmorrhage, which was the only symptom had existed four months when the disease proved fatal.

Case 2.—Cancer originating in the cervix of a uterus, the body of which was occupied by myomatous growths:—

Ada S., æt. 29, and married thirteen years, has had two children. The last child was born eight years ago. Menstruation, established at the age of sixteen, has recurred regularly, and the flow has usually lasted four days. She was unwell ten days before coming under observation. For four months she has noticed occasionally a blood-coloured discharge from the vagina, and for one week she has complained of difficulty in holding the water.

The abdomen is occupied by a firm irregular swelling which reaches to the umbilicus from the pubes. The vaginal roof is occupied by an irregularly nodulated and excavated mass. To the right of this mass is felt a portion of the abdominal tumour. The abdominal tumour is continuous with the cervix, and is the body of the uterus occupied by myomatous growths.

